# Circulating heat shock protein 90 (Hsp90) and autoantibodies to Hsp90 are increased in patients with atopic dermatitis

**DOI:** 10.1007/s12192-021-01238-w

**Published:** 2021-09-16

**Authors:** Krzysztof Sitko, Marta Bednarek, Jagoda Mantej, Magdalena Trzeciak, Stefan Tukaj

**Affiliations:** 1 Department of Molecular Biology, Faculty of Biology, University of Gdańsk, Wita Stwosza 59, 80-308 Gdańsk, Poland; 2 Department of Dermatology, Venerology and Allergology, Medical University of Gdańsk, Gdańsk, Poland

**Keywords:** Atopic dermatitis, AD, Allergy, IgE, Autoimmunity, Autoantibodies, Heat shock proteins, Hsps, Hsp90

## Abstract

Atopic dermatitis (AD) is one of the most common chronic inflammatory dermatoses characterized by persistent itching and recurrent eczematous lesions. While the primary events and key drivers of AD are topics of ongoing debate, cutaneous inflammation due to inappropriate IgE (auto)antibody–related immune reactions is frequently considered. Highly conserved and immunogenic heat shock protein 90 (Hsp90), a key intra- and extracellular chaperone, can activate the immune response driving the generation of circulating anti-Hsp90 autoantibodies that are found to be elevated in several autoimmune disorders. Here, for the first time, we observed that serum levels of Hsp90 and anti-Hsp90 IgE autoantibodies are significantly elevated (*p* < 0.0001) in AD patients (*n* = 29) when compared to age- and gender-matched healthy controls (*n* = 70). We revealed a positive correlation (0.378, *p* = 0.042) between serum levels of Hsp90 and the severity of AD assessed by Scoring Atopic Dermatitis (SCORAD). In addition, seropositivity for anti-Hsp90 IgE has been found in 48.27% of AD patients and in 2.85% of healthy controls. Although further studies on a larger group of patients are needed to confirm presented data, our results suggest that extracellular Hsp90 and autoantibodies to Hsp90 deserve attention in the study of the mechanisms that promote the development and/or maintenance of atopic dermatitis.

## Introduction

Atopic dermatitis (AD) is one of the most common chronic inflammatory skin diseases, with an incidence of 15–30% in children and 2–10% in adults that is characterized by intense itching and recurrent eczematous lesions. Current therapy of AD consists of topical emollients for cutaneous barrier dysfunction and topical corticosteroids or calcineurin inhibitors for skin inflammation. In severely affected cases, phototherapy, systemic immunosuppressants, and biologic agents are indicated. In addition, immunoglobulin E (IgE)–selective immunoadsorption is proposed (Kasperkiewicz et al. 2018; Langan et al. 2020; Weidinger and Novak 2016).

While the underlying events and key drivers of AD are subject to ongoing debate, there are at least two major and converging pathophysiological abnormalities of epidermal structure due to decreased filaggrin (FLG) expression and allergen-specific IgE- and/or autoreactive IgE-mediated dermatitis (Langan et al. 2020). Defects in the epidermal barrier lead to the penetration of the skin by allergens, activation of T helper 2 (Th2)–like cell polarization via Langerhans and dendritic cells, and IgE class switching (Guttman-Yassky et al. 2013; Varricchi et al. 2018). In addition, the activity of various other immune cells belonging to both innate and adaptive arm of immune responses including eosinophils and Th1, Th17, or Th22 cell populations appears to be critically involved in the initiation and the progression of AD, especially in European, American, and Asian populations (Czarnowicki et al. 2019; Weidinger and Novak 2016).

Highly conserved and intracellularly expressed Hsp90 (constituting 2–3% of the total cellular proteins) is a key molecular chaperone that plays an essential role in protein folding and the activation of its protein substrates (‘clients’) both inside and outside the cell. Hsp90 is responsible for biological activity of key signalling molecules, such as kinases, steroid receptors, cell cycle regulators, and transcription factors regulating various cellular processes. Extracellular Hsp90 with its chaperone activity is able to induce extracellular matrix remodelling via assisting in matrix metalloproteinase (MMP) activation. Hsp90 can also activate humoral immune responses driving to the generation of self-reactive antibodies to Hsp90 (Seclì et al. 2021; Tukaj and Węgrzyn 2016). In fact, these autoantibodies are found to be elevated in several autoimmune diseases (Tukaj 2020; Tukaj and Kaminski 2019).

Hsp90 became the focus of interest of scientists in the context of the development of autoimmune/inflammatory conditions, since it is upregulated in inflamed tissues and its presence in the extracellular space had been associated with the autoimmune process (Tukaj 2020; Tukaj and Kaminski 2019; Tukaj and Węgrzyn 2016). Moreover, since IgE-dependent immune reactions to self-proteins have been associated with AD (Roesner and Werfel 2019; Tang et al. 2012), searching for the autoantigens that may play an important role in the pathogenesis of AD is desired. This study aimed to determine serum levels of Hsp90 and anti-Hsp90 autoantibodies of the IgE, IgG, IgM, and IgA isotype in a cohort of patients with AD and in age- and gender-matched healthy controls.

## Materials and methods

### Patients and controls

Twenty-nine patients with atopic dermatitis (mean age: 25.86 ± 6.30; gender: 15 males and 14 females) (Table 1) and 70 age- and gender-matched healthy controls (mean age: 28.44 ± 8.81; gender: 31 males and 39 females) have been included in this study. Healthy volunteers who suffered from autoimmune, allergic, or any other skin disorder have been excluded from the study. The use of human biological material was approved by a bioethics committee at the regional medical chamber in Gdańsk (Poland), and written informed consents were performed in accordance with the Declaration of Helsinki.

**Table 1:** Characteristics of atopic dermatitis (AD) patients

No	Age	Gender	AD duration (years)	SCORAD	IgE (IU/ml)	Asthma	Allergic rhinitis	Allergic conjunctivitis
1	21	M	< 2	51.2	1323.1		+	
2	18	M	< 2	67	2794.3	+		+
3	10	F	≥ 2	84	1539.0	+		+
4	10	F	≥ 2	82	1264.6	+		+
5	53	F	≥ 2	80	2692.8			
6	41	F	≥ 2	19	84.5			
7	23	F	< 2	32.6	52.8		+	+
8	16	F	< 2	45	1120.3		+	
9	20	M	≥ 2	19	3283.0			
10	23	F	< 2	48.3	1674.3	+	+	
11	20	M	≥ 2	38	158.4			
12	17	F	< 2	32.4	1326.1	+	+	+
13	21	M	< 2	39.6	150.0		+	
14	13	F	< 2	42.2	395.8		+	+
15	20	F	≥ 2	86	1105.0	+	+	
16	15	M	< 2	34.8	1369.0			
17	25	F	≥ 2	70.4	1349.0	+		
18	59	M	≥ 2	70.4	1122.2	+		
19	18	M	< 2	48.3	1032.7		+	+
20	25	M	< 2	71.5	1296.9		+	
21	43	M	≥ 2	45.5	414.9			
22	31	F	≥ 2	68.5	1022.1	+	+	
23	27	M	< 2	43.3	1678.7		+	+
24	37	M	< 2	62	781.2		+	+
25	32	M	< 2	24	1221.7	+		
26	22	F	< 2	58.5	1935.1			
27	37	M	< 2	55.5	17.4			
28	28	M	< 2	40	1039.5			
29	25	F	< 2	43	834.5			

### Detection of circulating Hsp90

Hsp90 was evaluated in serum by commercially available HSP90α (human) ELISA kit (Enzo Life Science), following the manufacturer’s instructions.

### Detection of anti-Hsp90 antibodies

Levels of IgE, IgG, IgM, and IgA against human Hsp90 were evaluated in the serum samples by a home-made enzyme-linked immunosorbent assay (ELISA), as described previously (Mantej et al. 2019). Briefly, medium-binding 96-well plates were coated with commercially available full-length native human Hsp90 (Enzo Life Sciences) protein at a concentration of 0.5 μg/ml in 0.1 M bicarbonate buffer at 4 °C overnight. The wells were blocked with 1% bovine serum albumin (BSA) in phosphate-buffered saline (PBS) at room temperature (RT) for 1.5 h. After a washing step (three times with PBS containing 0.05% Tween 20), the sera were diluted (1:50–1:200) in PBS containing 0.1% BSA, added to the wells and were incubated at RT for 1.5 h. Plates were then incubated with horseradish peroxidase (HRP)–conjugated anti-human IgE (Abcam), anti-human IgG (Sigma), anti-human IgM (Abcam), or anti-human IgA (BioLegend) secondary antibodies diluted in PBS containing 0.1% BSA at RT for 1 h. The TMB substrate solution (Sigma) was used to visualize HRP enzymatic reaction and the reaction was stopped by adding 0.5 M H_2_SO_4_. Optical density measurements were performed at 450 nm with an ELISA plate reader (VICTOR Multilabel Plate Reader, PerkinElmer).

### Total IgE levels

Serum IgE levels were detected by commercially available ELISA kit performed according to the manufacturer’s instructions (Invitrogen).

### Statistical analysis

Statistical analyses were performed using the GraphPad Prism 5 software (San Diego, CA). Data was analysed by the Mann–Whitney *U* test or Spearman’s rank correlation test. *p* values less than 0.05 were considered significant.

## Results

### Serum levels of Hsp90 are significantly elevated in patients with AD

**Fig. 1:**
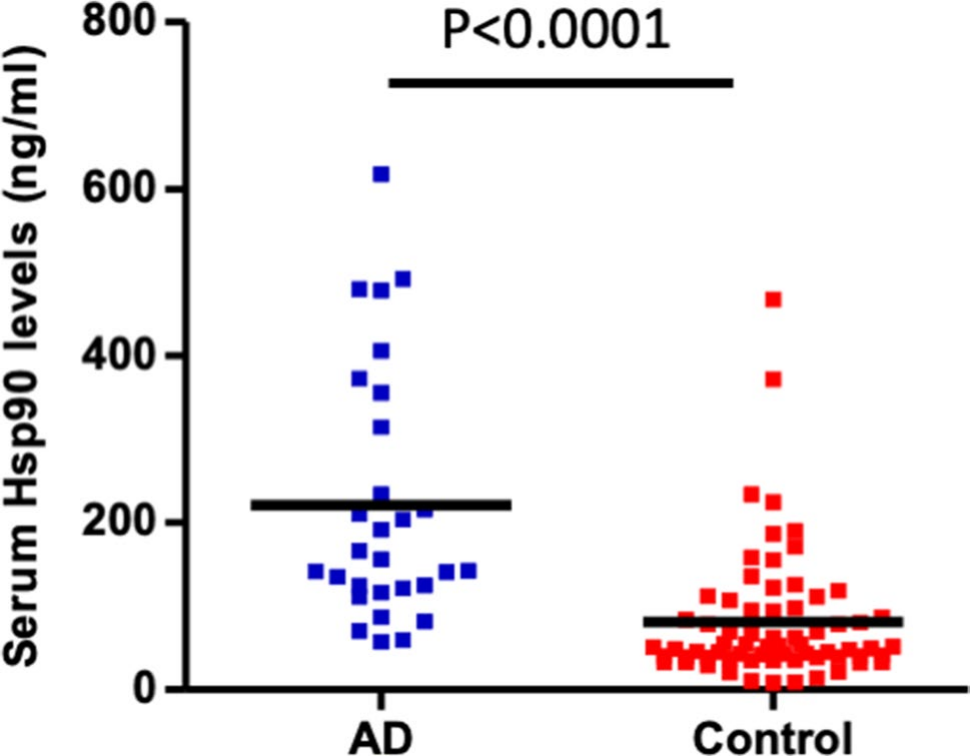
Levels of Hsp90 are increased in the sera of patients with atopic dermatitis (AD). Serum levels of Hsp90 in AD (*n* = 29) and age- and gender-matched healthy controls (*n* = 70) were assessed by an enzyme-linked immunosorbent assay. The dots and horizontal bars indicate individual and mean values in each group, respectively

In the analysis, 29 AD patients and 70 healthy controls were included. Clinical characteristics of the patients are stated in Table 1. Here, we found for the first time that serum levels of autologous Hsp90 were significantly elevated (2.79-fold increase; *p* < 0.0001) in AD patients when compared to age- and gender-matched healthy controls (mean ± SD; 215.41 ± 146.14 pg/ml vs. 84.64 ± 79.96 pg/ml), as measured quantitatively by a commercially available ELISA kit (Fig. 1).

### Serum levels of anti-Hsp90 IgE autoantibodies are increased in patients with AD

**Fig. 2:**
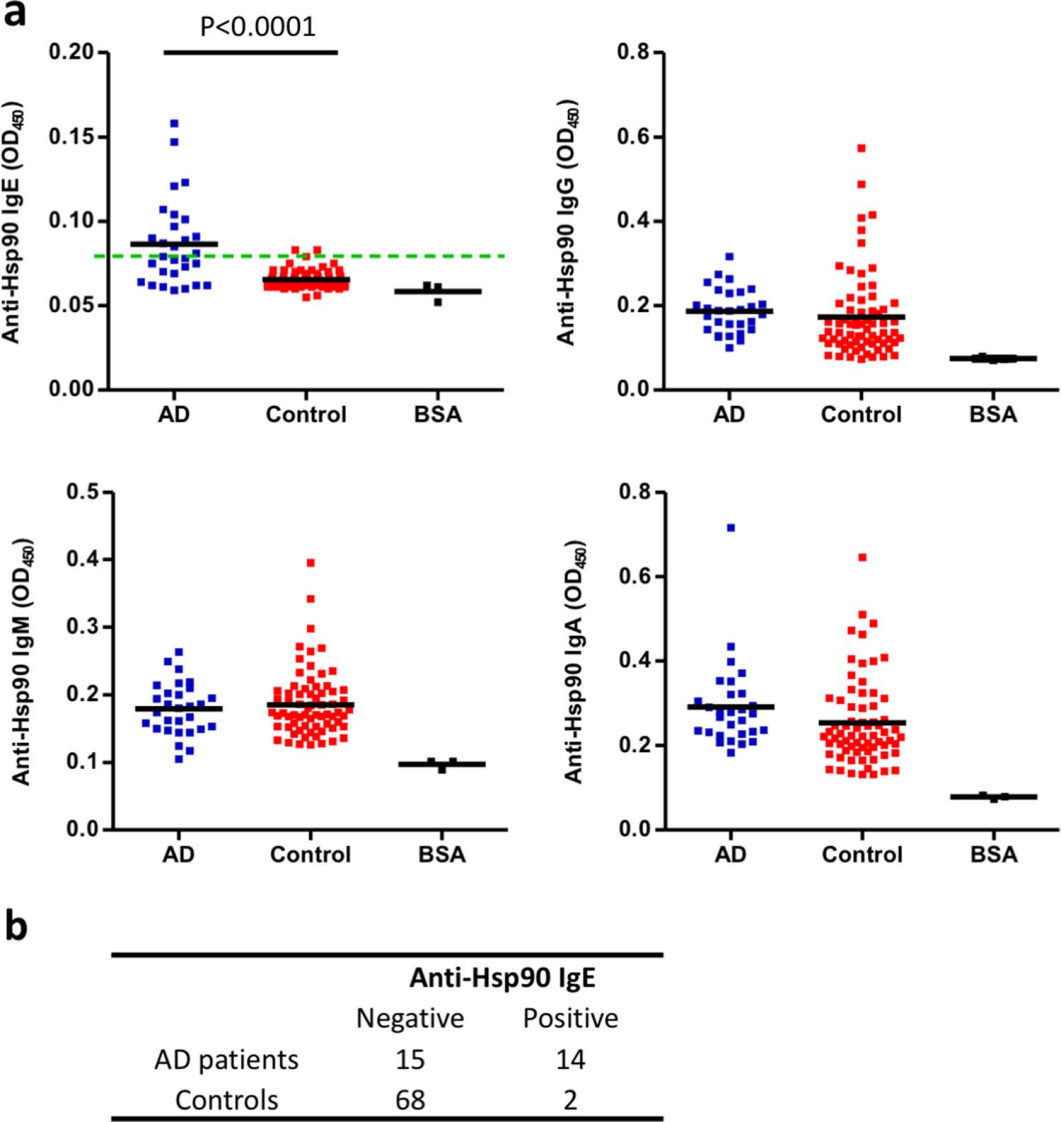
Circulating anti-Hsp90 IgE autoantibodies are increased in patients with AD. **a** Levels of anti-Hsp90 of the IgE, IgG, IgM, and IgA autoantibody isotype in the sera of AD patients (*n* = 29) and age- and gender-matched healthy controls (*n* = 70), measured by enzyme-linked immunosorbent assay. The optical density was measured at 450 nm (OD_450_). Green horizontal dashed line represents the cut-off value calculated as 3 × standard deviation above the mean of the control. The dots and horizontal bars indicate individual and mean values in each group, respectively. *p* values less than 0.05 were considered significant. **b** Contingency analysis showing anti-Hsp90 IgE positivity in AD patients and in healthy controls

Using home-made indirect ELISA assay, we found for the first time that serum levels of anti-Hsp90 IgE were significantly elevated (*p* < 0.0001) in AD patients (*n* = 29) as compared to age- and gender-matched healthy controls (*n* = 70), whereas levels of anti-Hsp90 IgG, IgM, or IgA were similar between both groups (Fig. 2a). Values of sera reactivity with Hsp90 measured above the mean values for bovine serum albumin (BSA) reactivity (negative control) were regarded as positive. In addition, taking into account the cut-off value calculated as 3 × standard deviation above the mean of the control, 14 out of 29 (48.27%) AD patients were anti-Hsp90 IgE positive, while only 2 out of 70 (2.85%) healthy controls matched such criteria (Fig. 2b).

### Serum levels of Hsp90 are associated with the clinical severity of AD

**Fig. 3:**
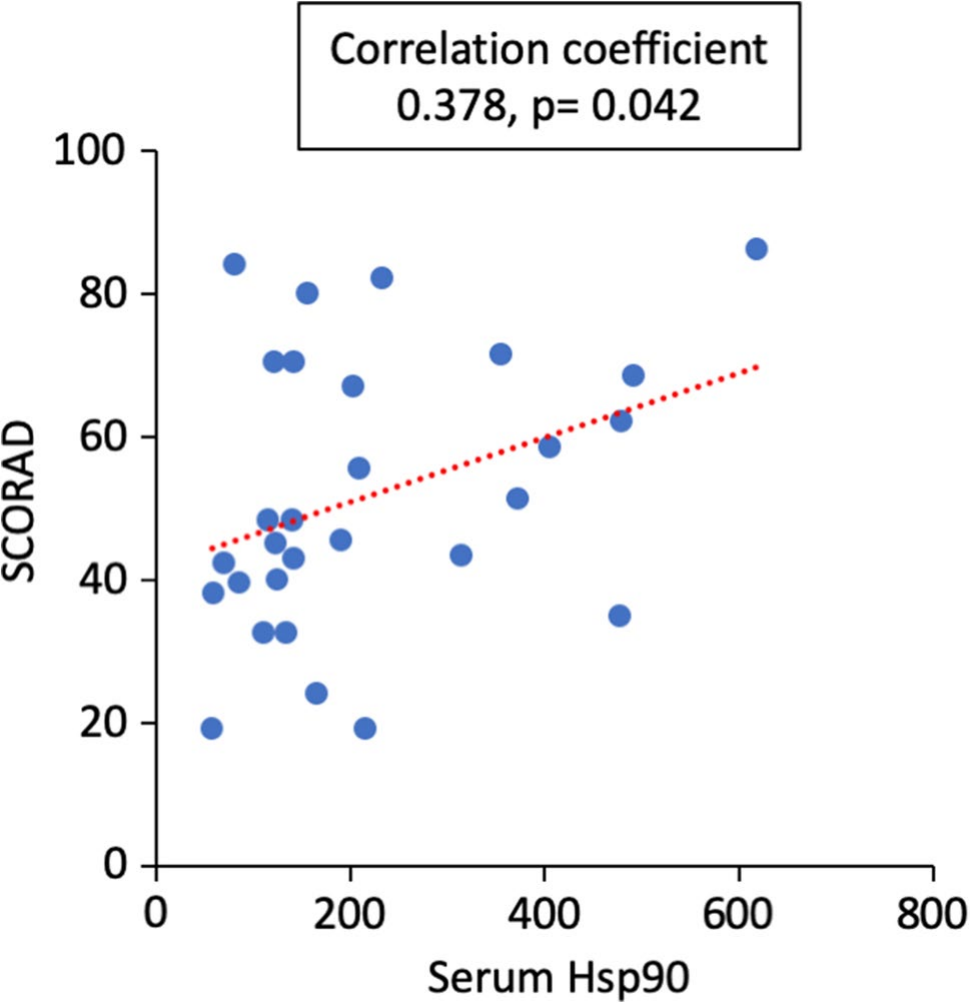
Higher serum levels of Hsp90 are associated with the clinical severity of AD. Correlation analysis between Hsp90 serum levels and the clinical severity (SCORAD)

Using Spearman’s rank correlation coefficient test, relationships between higher levels of circulating Hsp90 or anti-Hsp90 IgE autoantibodies and selected parameters of AD patients (Table 1) including disease activity, serum levels of IgE, or comorbidities were analysed. While a significant positive correlation (*r* = 0.378, *p* = 0.042) between serum levels of Hsp90 and the clinical severity of AD (SCORAD) was observed (Fig. 3), there were no significant associations between Hsp90 and serum levels of total IgE (0.317, *p* = 0.093) or anti-Hsp90 IgE (− 0.055, *p* = 0.776) and the presence of comorbidities such as asthma (0.043, *p* = 0.823), allergic rhinitis (0.091, *p* = 0.638), or allergic conjunctivitis (− 0.142, *p* = 0.461). Likewise, no significant correlations between anti-Hsp90 IgE serum levels and SCORAD (− 0.020, *p* = 0.914) as well as serum levels of total IgE (0.216, *p* = 0.258) and the presence of comorbidities such as asthma (− 0.086, *p* = 0.654), allergic rhinitis (− 0.074, *p* = 0.700), or allergic conjunctivitis (0.057, *p* = 0.765) could be recorded. Levels of either Hsp90 or anti-Hsp90 IgE were not dependent on the duration of AD (data not shown).

## Discussion

Heat shock proteins (Hsps) are a diverse group of molecules, either expressed constitutively or stress-induced, that are classified into several families based on their molecular weight and the characteristic structural domains. They mediate a range of essential cellular functions, including folding of newly synthesized polypeptides and renaturation or stabilization of biologically active proteins (Tukaj 2020; Tukaj and Kaminski 2019). Highly expressed Hsp90 is a key molecular chaperone that can be released to the extracellular milieu. Hsp90 facilitates the maturation of various ‘client’ proteins such as kinases, transcription factors, steroid hormone receptors, and E3 ubiquitin ligases that are involved in many different cellular pathways, including inflammation, growth, differentiation, and apoptosis (Schopf et al. 2017; Trepel et al. 2010). Since aberrant expression and secretion of the Hsp90 has been observed in cancer cells and chronically inflamed tissues, this chaperone has attracted scientists’ attention particularly in terms of development, progression, and treatment of cancer and autoimmune/inflammatory diseases (Tukaj 2020; Tukaj and Kaminski 2019; Tukaj et al. 2013, 2015b; Tukaj and Węgrzyn 2016). Here, for the first time, we found that serum levels of Hsp90 were significantly elevated in AD patients when compared to age- and gender-matched healthy controls and positively correlated with the clinical severity (SCORAD) of patients. While the pathological role of Hsp90 in AD needs to be further elucidated, we here propose non-exclusive explanations to the potential contribution of this chaperone in AD. It has been previously reported that MMPs are ‘client’ proteins of Hsp90 and dependent on its chaperone function (Seclì et al. 2021). As the interactions between MMPs and extracellular Hsp90 have been previously described in the context of tumour cell invasion and metastasis (Eustace et al. 2004; Garcia-Carbonero et al. 2013), we may speculate that elevated serum levels of Hsp90 could be involved in AD progression via promotion of MMP activation, the latter being known as an important pathophysiological factor in AD (Devillers et al. 2007; Harper et al. 2010).

The role of highly immunogenic, extracellular Hsp90 in AD may also be associated with its ability to activate the humoral (auto)immune response. Under stress conditions, Hsp90 can be released to the extracellular milieu and activate both the innate and adaptive immune responses driving the generation of circulating anti-Hsp90 autoantibodies that are found to be elevated in several autoimmune diseases, e.g., diabetes type 1 (Qin et al. 2003), systemic lupus erythematous (Ripley et al. 2001), rheumatoid arthritis (Mantej et al. 2019), dermatitis herpetiformis (Kasperkiewicz et al. 2014), and coeliac disease (Tukaj et al. 2017b). Here, for the first time, we found that serum levels of anti-Hsp90 IgE were significantly elevated in AD patients as compared to healthy controls, whereas levels of anti-Hsp90 IgG, IgM, or IgA were similar between both groups. It is well established that elevated serum levels of total IgE are found in about 80% of AD patients (Weidinger and Novak 2016). Here, seropositivity for anti-Hsp90 IgE has been found in about 50% of AD patients.

In the past, molecular mimicry hypothesis suggested that the immune response originally directed to the bacterial Hsps may be re-directed towards their human counterparts and in this way promote development of autoimmune reactions (Albani et al. 1995). While immune cross-reactions between foreign and self-antigens were experimentally confirmed in the case of Hsp40, Hsp60, or Hsp70 chaperones (Kotlarz et al. 2013; van Eden et al. 2017), clinical consequences of such immune cross-reactions have brought ambiguous outcomes (Tukaj and Kaminski 2019; Tukaj et al. 2021; van Eden et al. 2017). Information on a potential contribution of the bacterial Hsp90 (HtpG) in autoimmunity and the immune cross-reactions between HtpG and human Hsp90, however, is generally lacking. Since human Hsp90 shares about 40% sequence identity and 55% similarity with bacterial (*E. coli*) Hsp90 (Huai et al. 2005), immune cross-reactions between these molecules are theoretically possible. On the other hand, Kawano et al. (2004) found that set of antibodies raised against bacterial HtpG did not cross-react with all four human Hsp90 analogues, and vice versa. Therefore, we postulate that significantly higher levels of self-Hsp90 in the blood of AD patients led to the activation of the humoral autoimmune response and the production of anti-Hsp90 IgE autoantibodies. Since IgE-dependent immune reactions to self-proteins have been already associated with AD by many authorities on the matter (Roesner and Werfel 2019; Tang et al. 2012; Zeller et al. 2009), it is tempting to speculate that autoimmune reactions towards self-Hsp90 play an important role in the pathogenesis of AD. In fact, autoreactive IgE can also be found in other inflammatory skin diseases, such as autoimmune bullous diseases (AIBD). For example, patients suffering from the bullous pemphigoid, the most common form of AIBD, are IgG-, IgA-, or IgE-sensitized to the hemidesmosomal BP180 NC16A protein of the dermal–epidermal junction (Liu et al. 2017). The relevance of anti-Hsp90 IgE in AD, however, needs to be further examined since no significant associations between the levels of anti-Hsp90 IgE and total IgE or the clinical severity of AD were found in this study. On the other hand, lack of significant associations between anti-Hsp90 IgE and comorbidities (i.e., asthma, allergic rhinitis, or conjunctivitis) in AD patients may suggest a disease-specific IgE-dependent immune response to Hsp90 in AD.

Finally, numerous studies have shown that pharmacological inhibition of Hsp90 is successfully used in murine models of (auto)inflammatory diseases including AIBD via modulation of humoral and cellular immune responses (Kasperkiewicz et al. 2011; Tukaj et al. 2017a, 2015a, 2014a; Tukaj and Węgrzyn 2016; Tukaj et al. 2014b; 2015b), further supporting a potential role of this chaperone in the inflammatory process.

## Conclusions

Although further studies on a larger group of patients and additional experimental analysis are needed to confirm the present data, our results suggest that extracellular Hsp90 and autoantibodies to the Hsp90 deserve attention in the study of the mechanisms that promote the development and maintenance of atopic dermatitis.

## References

[CR1] Albani S, Keystone EC, Nelson JL, Ollier WER, La Cava A, Montemayor AC, Weber DA, Montecucco C, Martini A, Carson DA (1995). Positive selection in autoimmunity: abnormal immune responses to a bacterial dnaJ antigenic determinant in patients with early rheumatoid arthritis. Nat Med.

[CR2] Czarnowicki T, He H, Krueger JG, Guttman-Yassky E (2019). Atopic dermatitis endotypes and implications for targeted therapeutics. J Allergy Clin Immunol.

[CR3] Devillers AC, van Toorenenbergen AW, Klein Heerenbrink GJ, Muldert PG, Oranje AP (2007). Elevated levels of plasma matrix metalloproteinase-9 in patients with atopic dermatitis: a pilot study. Clin Exp Dermatol.

[CR4] Eustace BK, Sakurai T, Stewart JK, Yimlamai D, Unger C, Zehetmeier C, Lain B, Torella C, Henning SW, Beste G (2004). Functional proteomic screens reveal an essential extracellular role for hsp90α in cancer cell invasiveness. Nat Cell Biol.

[CR5] Garcia-Carbonero R, Carnero A, Paz-Ares L (2013). Inhibition of HSP90 molecular chaperones: moving into the clinic. Lancet Oncol.

[CR6] Guttman-Yassky E, Dhingra N, Leung DY (2013). New era of biologic therapeutics in atopic dermatitis. Expert Opin Biol Ther.

[CR7] Harper JI, Godwin H, Green A, Wilkes LE, Holden NJ, Moffatt M, Cookson WO, Layton G, Chandler S (2010). A study of matrix metalloproteinase expression and activity in atopic dermatitis using a novel skin wash sampling assay for functional biomarker analysis. Br J Dermatol.

[CR8] Huai Q, Wang H, Liu Y, Kim HY, Toft D, Ke H (2005). Structures of the N-terminal and middle domains of E. coli Hsp90 and conformation changes upon ADP binding. Structure.

[CR9] Kasperkiewicz M, Müller R, Manz R, Magens M, Hammers CM, Somlai C, Westermann J, Schmidt E, Zillikens D, Ludwig RJ (2011). Heat-shock protein 90 inhibition in autoimmunity to type VII collagen: evidence that nonmalignant plasma cells are not therapeutic targets. Blood.

[CR10] Kasperkiewicz M, Schmidt E, Ludwig RJ, Zillikens D (2018). Targeting IgE antibodies by Immunoadsorption in atopic dermatitis. Front Immunol.

[CR11] Kasperkiewicz M, Tukaj S, Gembicki A-J, Silló P, Görög A, Zillikens D, Kárpáti S (2014). Evidence for a role of autoantibodies to heat shock protein 60, 70, and 90 in patients with dermatitis herpetiformis. Cell Stress Chaperones.

[CR12] Kawano T, Kobayakawa T, Fukuma Y, Yukitake H, Kikuchi Y, Shoji M, Nakayama K, Mizuno A, Takagi T, Nemoto TK (2004). A comprehensive study on the immunological reactivity of the Hsp90 molecular chaperone. J Biochem.

[CR13] Kotlarz A, Tukaj S, Krzewski K, Brycka E, Lipinska B (2013). Human Hsp40 proteins, DNAJA1 and DNAJA2, as potential targets of the immune response triggered by bacterial DnaJ in rheumatoid arthritis. Cell Stress Chaperones.

[CR14] Langan SM, Irvine AD, Weidinger S (2020). Atopic dermatitis. Lancet.

[CR15] Liu Y, Li L, Xia Y (2017). BP180 is critical in the autoimmunity of bullous pemphigoid. Front Immunol.

[CR16] Mantej J, Polasik K, Piotrowska E, Tukaj S (2019). Autoantibodies to heat shock proteins 60, 70, and 90 in patients with rheumatoid arthritis. Cell Stress Chaperones.

[CR17] Qin H-Y, Mahon JL, Atkinson MA, Chaturvedi P, Lee-Chan E, Singh B (2003). Type 1 diabetes alters anti-hsp90 autoantibody isotype. J Autoimmun.

[CR18] Ripley BJ, Isenberg DA, Latchman DS (2001). Elevated levels of the 90 kDa heat shock protein (hsp90) in SLE correlate with levels of IL-6 and autoantibodies to hsp90. J Autoimmun.

[CR19] Roesner LM, Werfel T (2019). Autoimmunity (or not) in atopic dermatitis. Front Immunol.

[CR20] Schopf FH, Biebl MM, Buchner J (2017). The HSP90 chaperone machinery. Nat Rev Mol Cell Biol.

[CR21] Seclì L, Fusella F, Avalle L, Brancaccio M (2021). The dark-side of the outside: how extracellular heat shock proteins promote cancer. Cell Mol Life Sci.

[CR22] Tang TS, Bieber T, Williams HC (2012). Does "autoreactivity" play a role in atopic dermatitis?. J Allergy Clin Immunol.

[CR23] Trepel J, Mollapour M, Giaccone G, Neckers L (2010). Targeting the dynamic HSP90 complex in cancer. Nat Rev Cancer.

[CR24] Tukaj S (2020). Heat shock protein 70 as a double agent acting inside and outside the cell: insights into autoimmunity. Int J Mol Sci.

[CR25] Tukaj S, Bieber K, Kleszczyński K, Witte M, Cames R, Kalies K, Zillikens D, Ludwig RJ, Fischer TW, Kasperkiewicz M (2017). Topically applied Hsp90 Blocker 17AAG inhibits autoantibody-mediated blister-inducing cutaneous inflammation. J Invest Dermatol.

[CR26] Tukaj S, Görög A, Kleszczyński K, Zillikens D, Kárpáti S, Kasperkiewicz M (2017). Autoimmunity to heat shock proteins and vitamin D status in patients with celiac disease without associated dermatitis herpetiformis. The J Steroid Biochem Mol Biol.

[CR27] Tukaj S, Hellberg L, Ueck C, Hänsel M, Samavedam U, Zillikens D, Ludwig RJ, Laskay T, Kasperkiewicz M (2015). Heat shock protein 90 is required for ex vivo neutrophil-driven autoantibody-induced tissue damage in experimental epidermolysis bullosa acquisita. Exp Dermatol.

[CR28] Tukaj S, Kaminski M (2019). Heat shock proteins in the therapy of autoimmune diseases: too simple to be true?. Cell Stress Chaperones.

[CR29] Tukaj S, Kleszczyński K, Vafia K, Groth S, Meyersburg D, Trzonkowski P, Ludwig RJ, Zillikens D, Schmidt E, Fischer TW (2013). Aberrant expression and secretion of heat shock protein 90 in patients with bullous pemphigoid. PLoS ONE.

[CR30] Tukaj S, Mantej J, Sobala M, Potrykus K, Tukaj Z, Zillikens D, Ludwig RJ, Bieber K, Kasperkiewicz M (2021). Therapeutic implications of targeting heat shock protein 70 by immunization or antibodies in experimental skin inflammation. Front Immunol.

[CR31] Tukaj S, Tiburzy B, Manz R, de Castro Marques A, Orosz A, Ludwig RJ, Zillikens D, Kasperkiewicz M (2014). Immunomodulatory effects of heat shock protein 90 inhibition on humoral immune responses. Exp Dermatol.

[CR32] Tukaj S, Węgrzyn G (2016). Anti-Hsp90 therapy in autoimmune and inflammatory diseases: a review of preclinical studies. Cell Stress Chaperones.

[CR33] Tukaj S, Zillikens D, Kasperkiewicz M (2014). Inhibitory effects of heat shock protein 90 blockade on proinflammatory human Th1 and Th17 cell subpopulations. J Inflamm (Lond).

[CR34] Tukaj S, Zillikens D, Kasperkiewicz M (2015). Heat shock protein 90: a pathophysiological factor and novel treatment target in autoimmune bullous skin diseases. Exp Dermatol.

[CR35] van Eden W, Jansen MAA, Ludwig I, van Kooten P, van der Zee R, Broere F (2017). The enigma of heat shock proteins in immune tolerance. Front Immunol.

[CR36] Varricchi G, Pecoraro A, Marone G, Criscuolo G, Spadaro G, Genovese A, Marone G (2018). Thymic stromal lymphopoietin isoforms, inflammatory disorders, and cancer. Front Immunol.

[CR37] Weidinger S, Novak N (2016). Atopic dermatitis. Lancet.

[CR38] Zeller S, Rhyner C, Meyer N, Schmid-Grendelmeier P, Akdis CA, Crameri R (2009). Exploring the repertoire of IgE-binding self-antigens associated with atopic eczema. J Allergy Clin Immunol.

